# The incidence and severity of errors in pharmacist-written discharge medication orders

**DOI:** 10.1007/s11096-017-0468-9

**Published:** 2017-06-01

**Authors:** Raliat Onatade, Sara Sawieres, Alexandra Veck, Lindsay Smith, Shivani Gore, Sumiah Al-Azeib

**Affiliations:** 1 Institute of Pharmaceutical Sciences, King’s College London, London, UK; 2 Pharmaceutical Sciences Clinical Academic Group, King’s Health Partners, London, UK; 3 Pharmacy Department, Barts Health NHS Trust, London, E1 2ES UK; 4 Pharmacy Department, King’s College Hospital NHS Foundation Trust, London, UK; 5 Pharmacy Department, Western Sussex Hospitals NHS Foundation Trust, Chichester, UK; 6 Pharmacy Department, Royal Brompton and Harefield NHS Foundation Trust, London, UK

**Keywords:** Hospital pharmacy, Medication, Medication errors, Medication safety, Patient discharge, Pharmacist, Prescribing, Quality, United Kingdom

## Abstract

*Background* Errors in discharge prescriptions are problematic. When hospital pharmacists write discharge prescriptions improvements are seen in the quality and efficiency of discharge. There is limited information on the incidence of errors in pharmacists’ medication orders. *Objective* To investigate the extent and clinical significance of errors in pharmacist-written discharge medication orders. *Setting* 1000-bed teaching hospital in London, UK. *Method* Pharmacists in this London hospital routinely write discharge medication orders as part of the clinical pharmacy service. Convenient days, based on researcher availability, between October 2013 and January 2014 were selected. Pre-registration pharmacists reviewed all discharge medication orders written by pharmacists on these days and identified discrepancies between the medication history, inpatient chart, patient records and discharge summary. A senior clinical pharmacist confirmed the presence of an error. Each error was assigned a potential clinical significance rating (based on the NCCMERP scale) by a physician and an independent senior clinical pharmacist, working separately. *Main outcome measure* Incidence of errors in pharmacist-written discharge medication orders. *Results* 509 prescriptions, written by 51 pharmacists, containing 4258 discharge medication orders were assessed (8.4 orders per prescription). Ten prescriptions (2%), contained a total of ten erroneous orders (order error rate—0.2%). The pharmacist considered that one error had the potential to cause temporary harm (0.02% of all orders). The physician did not rate any of the errors with the potential to cause harm. *Conclusion* The incidence of errors in pharmacists’ discharge medication orders was low. The quality, safety and policy implications of pharmacists routinely writing discharge medication orders should be further explored.

## Impact on practice

- Pharmacists can safely write discharge medication orders as part of a routine clinical pharmacy service.
- Larger studies are needed to research the clinical significance of pharmacists’ medication order errors.
- Pharmacists writing discharge medication orders may offer opportunities to improve the quality and safety of patient care transitions.

## Introduction

Errors associated with hospital discharge prescriptions (To Take Aways, TTAs) are problematic and well documented in the UK [1–5]. For example, results from the EQUIP study of prescribing errors in twenty UK hospitals detected errors in 6.3% of doctors’ discharge medication orders (individual items in a prescription) [1]. In a study of prescribing errors in three UK mental health hospitals, 6.5% of discharge medication orders were associated with an error [5].

Accurate discharge medication orders are essential to ensure patient safety during transitions of care [2, 5]. Discrepancies can have important clinical consequences. Subsequent caregivers may also base actions and decisions on the information contained in discharge prescriptions, leading to inappropriate treatment if this information is inaccurate [2, 5].

Pharmacists are often cited as an essential defence in preventing prescribing mistakes reaching the patient [1, 3, 5–7] by detecting and correcting errors before medication administration. In 2003, suitably trained pharmacists were given the rights to prescribe in accordance with pre-agreed, condition-specific treatment plans. In 2006, this was extended and independent prescribing rights were granted, on completion of additional training [8, 9]. Non-prescribing pharmacists writing discharge medication orders instead of physicians has previously been reported, with noted improvements in quality and efficiency [10–13]. A North American study evaluating a pilot programme comparing physician- and pharmacist-written discharge medication prescriptions, found that pharmacists entered discharge medication orders more accurately than physicians. Pharmacists reviewed the physician-entered orders, and physicians reviewed the pharmacists’ orders from the intervention group. If a change was required to either the dose or frequency, an order was deemed inaccurate. Ninety-six per cent of pharmacists’ orders were accurate compared to 56% of physicians’ orders [10]. Although the authors emphasise that statistical analyses were not conducted, post hoc calculations indicate a significant difference between the two groups (95% CI for the difference in proportions = 33–46%, Chi Square test *p* < 0.0001). A study carried out in the UK compared pharmacist- and doctor-written discharge prescriptions on a surgical ward. Pharmacist-written prescriptions contained considerably fewer errors, omissions and unclear information in comparison to those written by doctors. All prescriptions were checked by the other profession, and additionally by clinical dispensary pharmacists. The number of dispensary pharmacists’ interventions increased 10-fold when checking doctor-written prescriptions. Additionally, doctors made 10 minor alterations to the pharmacists’ prescriptions, while pharmacists had to clarify the doctors’ prescriptions on 52 occasions [12]. Whilst demonstrating the value of pharmacists ordering discharge medication, these studies have only evaluated pilot programmes, on one or two hospital wards within an organisation. They included relatively few prescriptions or patients and the clinical significance of pharmacist errors was not evaluated.

### Aim of the study

The aim of this study was to quantify, describe and determine the clinical relevance of errors in pharmacist-written discharge medication orders in a large teaching hospital where pharmacists writing discharge medication orders is a routine clinical pharmacy service.

### Ethics approval

The study was approved by the local Research and Audit Committee as a retrospective service evaluation. Ethics approval was not required, in accordance with National Health Service Research Authority Guidelines.

## Method

### Study design

This was a retrospective, observational study.

### Definitions

Prescribing errors were defined according to Dean et al. [14]. A discharge prescription (TTA or PTTA if written by a pharmacist) is the list of medicines that a patient should be taking on discharge. A discharge medication order is an individual item within the list of medicines.

### Setting and participants

The study was undertaken in a 1000-bed teaching hospital in London, UK. Around 2600–3000 TTAs are processed every month by the hospital pharmacy. Pharmacists generate approximately 24,000 discharge medication orders every month, constituting 75–80% of all discharge medication orders. Electronic prescribing and medication administration (EPMA) is implemented across the hospital and discharge medication is ordered on the electronic system. There is no computerised decision support in the EPMA system. Pharmacists review all medication orders and discuss and resolve potential and actual errors with prescribers.

Once informed of a pending discharge, the pharmacist takes responsibility for writing the discharge medication orders. Medication for discharge is always supplied by the hospital pharmacy. The process of writing a list of discharge medication (pharmacist-written TTA, PTTA) using EPMA involves reordering the required inpatient medication as discharge medication orders. The pharmacist also consults with the patient, nurse and physician and checks the documented medication history. Pre-admission medication withheld during admission is restarted if appropriate and new medication may be started. The pharmacist sends a printed copy of the PTTA to the dispensary for medication to be supplied. Not all medication requires dispensing, as the patient may have sufficient supplies at home. However, all should be listed on the PTTA, for clarity. The dispensed medication is sent to the ward, and the printed list is retained in pharmacy. The pharmacists are writing discharge medication orders but they are not prescribing. At any point during this process, the patient’s physician checks the discharge medication orders, and completes, prints and signs the discharge notification (a larger document incorporating the pharmacist’s medication orders plus clinical and other treatment details), for the patient to take home with their medication. If any changes to the medication orders are required before discharge, the physician makes the changes, or the pharmacist will be notified by the physician or nurse to amend the PTTA. If the pharmacist makes the amendments, the PTTA will not be rechecked by another pharmacist. Any errors corrected at this stage would not have been identified as part of this study. An electronic copy of the discharge notification (eDN) is also sent to the patient’s general practitioner.

A sample size calculation was performed, using in-house exploratory data, collected prior to implementation of EPMA (i.e. when paper charts were in use). This indicated that 32% of physician-written TTAs contained at least one erroneous discharge medication order and 9% of all discharge medication orders were erroneous (9% order error rate). This determined that 500 PTTAs would be sufficient to observe an error rate similar to, or smaller than that found in physician-written TTAs. Stratified sampling was applied to ensure proportionate representation of wards or units belonging to each major clinical specialty. As a pharmacist will consistently work on just one or two wards, this also ensured orders made by a wide range of pharmacists were captured.

### Data collection

On convenient (when researchers were available) days between October 2013 and January 2014, researchers (AV, LS and SG—all pre-registration pharmacists who do not write PTTAs) reviewed all PTTAs dispensed by pharmacy one week earlier. The days chosen varied between Monday and Saturday. Data collection continued until the target sample sizes for the total number of PTTAs and the required number per specialty were reached. The printed PTTAs retained in the dispensary contain the pharmacist-written discharge medication orders, prior to the physician check. AV, LS and SG compared the PTTAs to three sources to detect errors—medication history, the inpatient drug chart and the eDN. The eDN is an unalterable document, authorised by the physician to be saved electronically to the patient record, after checking the medication orders and completing the clinical details. On both the PTTA and the eDN, the discharge medication orders have the name of the ordering professional listed alongside the order and the date any modifications were made. This made it possible to identify changes made by physicians after the pharmacist had sent the printed PTTA to the dispensary. If differences or discrepancies (including the addition or omission of a medication, duplication of therapy, differences in formulations, doses or dose frequencies) between the medication orders on the PTTA and any of the other sources were identified, all documentation was passed to a senior clinical pharmacist (SS or SA) who reviewed the patient record and used clinical judgment to decide if an error had occurred. SS would be given any PTTAs written by SA, and vice versa. Where necessary, the pharmacist who wrote the PTTA was asked to clarify a discrepancy. If an error was identified, the research team took appropriate remedial action, to ensure patient safety. Exclusions were any PTTAs which were checked by a second pharmacist before being dispensed (this would be the case when a pharmacist was being trained to write discharge medication orders), and any for which the comparative sources were missing or inaccessible.

Errors were categorised according to the type of prescription error (omission, commission/addition, duplication, administration frequency, dosage form, route) derived from those used in similar studies [1, 15, 16]. All errors were also independently rated for their potential clinical impact by one senior physician and one senior clinical pharmacist (not otherwise associated with the study). The raters were given descriptions of the errors (Table 2) and asked to use their clinical and professional judgment to categorise each error according to a validated adaptation of the National Coordinating Council for Medication Error Prevention (NCCMERP) index and descriptors for potential harm [17, 18]. Consensus between the two raters was not sought.

### Statistical analysis

Data were organised with Microsoft Excel 2011. Statistical analyses, including frequencies and proportions, were performed using IBM SPSS for Macintosh, Version 21.

## Results

Data collection took place over twenty-two days. A total of 509 PTTAs (509 patients), with 4258 discharge medication orders made by 51 pharmacists, were assessed—a mean of 8.4 orders per PTTA. This equated to approximately five days of PTTA workload. The breakdown of specialties is in Table 1. Overall, 10 errors in 10 PTTAs were detected, giving a 2% (10/509, 95% CI 0.8–3.2%) PTTA error rate. The percentage of orders with an error (order error rate) was 0.2% (10/4258, 95% CI 0.1–0.3%).

**Table 1:** Specialty breakdown of pharmacist-written discharge prescriptions (PTTAs)

Ward or unit specialty	Number of PTTAs	Percentage of total (%)
Acute medicine	175	34.4
Surgery	104	20.4
Cardiovascular	49	9.6
Paediatrics	45	8.8
Neurosciences	43	8.5
Liver	37	7.3
Haematology	19	3.7
Renal	17	3.3
Private patients (mixed specialties including neurosurgery, liver and general surgery)	11	2.2
Gynaecology	7	1.4
Obstetrics	2	0.4
Total	509	100

Table 2 gives details of each error. Overall agreement for harm versus no harm between the physician and pharmacist was 90%. As there were only ten errors, agreement was not corrected for chance. The physician did not rate any errors with the potential to cause harm. The pharmacist rated one error with the potential to cause temporary harm. This was an omission of a diabetic patient’s regular anti-hypoglycaemic medication from the PTTA. The patient had been using these before admission. This equated to 0.02% (1/4258) of pharmacist discharge medication orders potentially causing harm.

**Table 2:** Frequency of error types and harm categories

		Adapted NCCMERP category*
*Drug omitted—9/10 (90%)*		
1	An adolescent patient with a history of cystic fibrosis was using prescribed salbutamol inhaler as required, before and during admission. This was not listed as discharge medication. (Specialty—Paediatrics)	Physician: C Pharmacist: C
2	A child who had just undergone a liver transplant was using a combination asthma inhaler, before and during admission, but this was not listed as discharge medication. (Specialty—Paediatrics)	Physician: C Pharmacist: C
3	Oral glucose gel 40% and SC/IM glucagon to be used as required for hypoglycaemia were documented on a child’s pre-admission medication history but not listed as discharge medication. (Specialty—Paediatrics)	Physician: A Pharmacist: D
4	A patient, admitted for elective surgery, was taking lansoprazole before admission, but the indication was not known. During admission, this was changed to intravenous omeprazole, and later oral omeprazole with a stated indication of stress ulcer prophylaxis. Neither lansoprazole nor omeprazole were ordered as discharge medication. (Specialty—Liver)	Physician: C Pharmacist: C
5	A patient who had undergone elective liver surgery had been taking regular paracetamol prior to discharge however, this was not ordered on discharge. (Specialty—Liver)	Physician: C Pharmacist: C
6	A patient who had undergone an elective neurosurgical procedure had received a few doses of cyclizine whilst just before discharge and so should have been given a short course on discharge, however they were not. (Specialty—Neurosurgery)	Physician: C Pharmacist: C
7	A patient admitted with a fall, with a past medical history of peripheral vascular disease and type 2 diabetes mellitus was prescribed Capsaicin cream 1% to be applied to affected areas as required, before and during admission. This was not ordered as discharge medication. (Specialty—Acute Medicine)	Physician: C Pharmacist: C
8	A patient who had undergone elective orthopaedic surgery was taking an average of 40 mg morphine daily when required, plus regular paracetamol and tramadol in the three days prior to admission, but was not discharged with morphine. (Specialty—Surgery)	Physician: C Pharmacist: C
9	Omeprazole 20 mg daily was prescribed for a patient with a history of sickle cell disease, for epigastric pain during admission but was omitted from the discharge medication (Specialty—Haematology)	Physician: C Pharmacist: C
*Duplicate therapy—1/10 (10%)*		
1	A patient was started on trimethoprim at discharge, for a urinary tract infection whilst they were already on amoxicillin. There was no suggestion of resistance to amoxicillin. (Specialty—Surgery)	Physician: C Pharmacist: C

* Key A = Circumstances or events that have the capacity to cause error. C = The error would not cause patient harm OR the error would have required monitoring or intervention to confirm that it resulted in no harm. D = The error would likely have resulted in temporary harm to the patient and would have required intervention, initial hospitalization or prolonged hospitalization

## Discussion

This is the first study to quantify error rates in pharmacists’ discharge medication orders, where this is a routinely provided clinical pharmacy service. It is observational only, with no comparisons with physician error rates before pharmacists started writing the orders. The introduction of the pharmacy service coincided with EPMA implementation. Thus any attempt to draw conclusions from a direct before-and-after comparison of pharmacist- and physician-errors would be inappropriate.

The proportion of PTTAs containing an error was 2%, with 0.2% of all orders being erroneous. Franklin and colleagues in their study of doctors’ prescribing errors on UK medical and surgical wards, found that 9% of discharge medication orders from medical admissions and surgical wards were erroneous [3]. In a study of prescribing errors in nine hospitals in North-West England, Seden et al. [4] reported that 34.5% of TTAs written by a doctor contained at least one prescribing error. Studies of errors in pharmacists’ medication orders have not been widely reported. Baqir et al. [19] found an error rate of 0.3% in 1415 pharmacists’ medication orders for hospital inpatients. There is currently no equivalent information on pharmacists’ discharge prescriptions with which to compare our results. Additionally, we assessed pharmacists’ discharge medication orders in the context of an electronic prescribing and administration system, whereas the aforementioned studies used paper-based systems. The pharmacists’ error rate in this study was lower than that of physicians, found in a UK study of pharmacists’ interventions in physician-written discharge medication orders [16]. This study was also in the context of an electronic prescribing system. Orders were entered for 1038 patients. At least one error was found in 20.4% of discharge prescriptions. There were 630 erroneous orders out of a total of 7920 orders, an order error rate of 8%. Errors were rated as serious (2.9%), significant (76.3%) and minor (20.8%).

Independent raters found that the few pharmacists’ errors in this study had low clinical importance. These results and work by other researchers [19] indicates that much larger datasets are needed to draw conclusions on the potential for harm from pharmacist prescribing errors.

The comprehensive nature of this study is a significant strength. Unlike other work, we assessed prescriptions written by a wide range of pharmacists, working in all major clinical specialties. Therefore, a degree of real-world generalisation to similar hospitals with similar systems is possible; although electronic prescribing systems with clinical decision support may potentially further reduce errors. This study has also added to the emerging evidence regarding the safety of pharmacist prescribing [19].

There are some limitations to this work. Workflow constraints meant that it was not possible to check the PTTAs immediately after they were written. This reduced any potential Hawthorne effect (where individuals modify or improve their work because they are aware they are being observed). However, corrections requested by physicians before the PTTA was printed and changes made by physicians, but not documented, will not have been detected. Changes made by dispensary staff to orders on the printed PTTA will also not have been identified. However, our method for identifying errors in PTTAs matched the process pharmacists use when checking physician-written TTAs (i.e. reconciling the TTA with the medication history, inpatient drug chart and information in the patient record). Therefore, it is not likely that there were sufficient undetected errors to have had a significant effect on the outcome. Due to restricted researcher availability, data collection occurred on conveniently selected days. It was felt that ensuring proportionate representation of all the major clinical specialties was more methodologically important than randomisation. Risk of bias due to non-randomisation was minimised by ensuring that only the researchers knew the data collection days in advance and by varying the chosen day of the week. Additionally, the ordering of discharge medication by pharmacists is a routine service in the organisation and there were no interruptions to the service during the study. Therefore, there was little possibility of differences in activity between data collection and non-data collection days. An additional bias may have been introduced by excluding PTTAs for which all three comparative sources could not be found.

The system of pharmacists writing discharge medication orders, as described above, is not considered prescribing and the orders have to be checked and signed by a physician. However, pharmacists have taken over a significant role which used to be the sole domain of physicians. A full discussion of the potential negative impact of this type of role change on physicians’ opportunities to develop prescribing skills and learn from their own prescribing errors [20, 21] is outside the scope of this work, and should be the subject of future research.

The roles pharmacists can play in improving patient safety at care transitions are well documented [22, 23]. Activities mainly encompass medication reconciliation [24–26], patient education programmes and post-discharge follow-up [22, 23, 27–29] and medication reviews [22, 23, 27]. By demonstrating that pharmacists can safely write discharge medication orders, the present study has expanded the evidence-base regarding pharmacist-led discharge activities.

Further studies are needed, including direct comparisons with medical and other non-medical prescribers, and in other settings, in order to explore this new role for pharmacists in improving the quality and safety of care transitions.

## Conclusion

In this study, the incidence of errors in pharmacist-written discharge medication orders was 0.2%. The majority of errors were omitted medications, with one incidence of duplicated therapy. The clinical relevance of the errors was minimal although firm conclusions cannot be drawn because of the low number of errors. There are well-known safety and quality issues with traditional physician-written discharge prescriptions, therefore the policy implications of our findings are important.
